# Contribution of Case Reports to Brain Metastases Research: Systematic Review and Analysis of Pattern of Citation

**DOI:** 10.1371/journal.pone.0034300

**Published:** 2012-03-28

**Authors:** Carsten Nieder, Adam Pawinski, Astrid Dalhaug

**Affiliations:** 1 Department of Oncology and Palliative Medicine, Nordland Hospital, Bodø, Norway; 2 Institute of Clinical Medicine, Faculty of Health Sciences, University of Tromsø, Tromsø, Norway; Aalto University, Finland

## Abstract

Research activity related to different aspects of prevention, prediction, diagnosis and treatment of brain metastases has increased during recent years. One of the major databases (Scopus) contains 942 scientific articles that were published during the 5-year time period 2006–2010. Of these, 195 (21%) reported on single patient cases and 12 (1%) were reports of 2 cases. Little is known about their influence on advancement of the field or scientific merits. Do brain metastases case reports attract attention and provide stimuli for further research or do they go largely unrecognized? Different measures of impact, visibility and quality of published research are available, each with its own pros and cons. For the present evaluation, article citation rate was chosen. The median number of citations overall and stratified by year of publication was 0, except for the year 2006 when it was 2. As compared to other articles, case reports remained more often without citation (p<0.05 except for 2006 data). All case reports with 10 or more citations (n = 6) reported on newly introduced anticancer drugs, which commonly are prescribed to treat extracranial metastases, and the responses observed in single patients with brain metastases. Average annual numbers of citations were also calculated. The articles with most citations per year were the same six case reports mentioned above (the only ones that obtained more than 2.0 citations per year). Citations appeared to gradually increase during the first two years after publication but remained on a generally low or modest level. It cannot be excluded that case reports without citation provide interesting information to some clinicians or researchers. Apparently, case reports describing unexpected therapeutic success gain more attention, at least in terms of citation, than others.

## Introduction

Development of brain metastases is a common problem in oncology [1]. Given the large number of patients and important consequences for individual patients and health care systems, intense research activity is directed towards prevention and therapy. Landmark phase III randomized trials, other prospective studies, improved multidisciplinary interaction, and technology improvement provided the framework for recent treatment advances. If one looks at all scientific publications a broad mix of prospective and retrospective studies, reviews, and case reports can be identified. Intuitively, one would expect at best minor or moderate influence of small retrospective studies or even case reports on changes in clinical practice. However, little is known about the actual attention that case reports receive after publication and whether or not they are cited by other publications. Measuring their impact is not trivial. Impact factor of journals that publish case reports is a two-edged sword, e.g. regarding its correlation with the true scientific or practical impact of the reports [2]–[6]. Article download rates might provide some indication for visibility and impact, but will depend on presence and quantity of fees charged by the publisher. Another potential measure of quality and impact of research is the citation rate [7]–[10]. Landmark or practice-changing research is likely to be cited by successor trials, editorials, review articles, meta-analyses and guidelines. In our attempt to evaluate the role of case reports in brain metastases therapy, including related areas of diagnostic and prevention, we therefore relied on citation rates of such reports that were published between 2006 and 2010. We hypothesized that case reports might have accumulated fewer citations than other articles.

## Methods

A systematic search of the abstract and citation database Scopus (Elsevier B.V., www.scopus.com) by use of the key words ‘brain metastases’, ‘cerebral metastases’, ‘intracranial metastases’, ‘central nervous system metastases’ or ‘secondary brain tumor’ was performed on November 28^th^ and 29^th^ 2011. To begin with, publications related to metastases from extracranial solid tumors in pediatric and adult patients were selected irrespective of language and article type (case report, review, randomized trial, meta-analysis etc.). In other words, all epidemiologic, diagnostic, therapeutic and preclinical topics published in the time period 2006–2010 were included. The issues of prophylactic cranial irradiation and leptomeningeal carcinomatosis were not included unless for example an article covered both leptomeningeal and parenchymal brain metastases. Articles dealing with brain metastases and glioma, e.g. related to differential imaging diagnosis, were included as well. Then, all case reports describing one or two patients were extracted and patterns of citation (field ‘times cited’ in the Scopus database) were evaluated. Since the title of an article might or might not indicate that one or two cases are reported, we accessed abstracts or if necessary complete articles to make sure that all eligible publications were included. We evaluated median number of citations, proportion of case reports without citation, total number of citations accumulated independently of their origin, and average annual number of citations. Statistical comparisons were performed with the Fisher exact probability and Mann-Whitney U tests. A one-sided p-value<0.05 was considered statistically significant.

## Results

**Figure 1:**
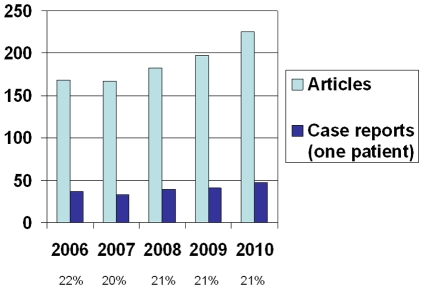
Number of articles and case reports published per year.

Overall 942 brain metastases publications were identified (167–226 per year). Of these, 195 (21%) reported on single patient cases and 12 (1%) were reports of 2 cases (a complete list can be requested from the corresponding author). Figure 1 shows the numbers of publications per year. Overall, publication numbers have increased in the time period 2006–2010. The proportion of case reports remained constant. Table 1 shows the citation patterns. The median number of citations of non-case-reports published in a given year was lower in 2009 and 2010 as compared to previous years. In other words, accumulation of citations takes time. Case reports were cited less often. Their median number of citations overall and stratified by year was 0, except for the year 2006. As compared to other articles, case reports remained more often without citation (p<0.05, except for 2006 data). Table 2 shows the most cited case reports overall [11]–[16], arbitrarily defined as 10 or more citations (n = 6). All of these were published in English language and reported on newly introduced anticancer drugs, which commonly are prescribed to treat extracranial metastases, and the responses observed in single patients with brain metastases. All were published before 2009. Since articles published for example in 2006 are more likely to have accumulated a large number of citations than articles published in 2010, average annual numbers of citations were also calculated. For this purpose, 2011 was defined as 0.92 years (11 of 12 months; January–November). The articles with most citations per year are also shown in Table 2. They were identical to the 6 publications with 10 or more citations and had 2.5–6.1 citations per year. All others had less than 2.0 citations per year. Group authorship was most common. Five case reports (2.4%) had one author, 12 (5.8%) had two authors, 26 (12.6%) had 3 authors, 39 (18.9%) had four authors, and the others (60.3%) had more than four authors.

**Table 1:** Citation patterns: Statistically significant differences between case reports (one patient) and other articles were seen regarding median number (except for 2010 data) and percent without citation (except for 2006 data).

	2006	2007	2008	2009	2010
*Median number of total citations, range (other articles)*	5, 0–282	5, 0–89	4, 0–108	3, 0–123	1, 0–42
*Median number of total citations, range (case reports, one patient)*	2, 0–15	0, 0–30	0, 0–12	0, 0–4	0, 0–2
*Median number of total citations, range (case reports, two patients)*	3, 0–4	1*	0*	0, 0–2	0, 0–2
*Percent without citation (other articles)*	20	23	27	26	40
*Percent without citation (case reports, one patient)*	27	52	51	61	68
*Percent without citation (case reports, two patients)*	33	0*	100*	67	75

* only one published article.

Because only 12 case reports on two patients were available, no statistical tests were performed for this subgroup.

**Table 2:** Case reports with most citations, i.e. > = 10 (absolute count; all articles reported on single cases).

*Authors and year of publication*	*Title*	*Absolute citation count*	*Citations per year*
Medioni et al. 2007 [11]	Complete cerebral response with sunitinib for metastatic renal cancer	30	6.1
Koutras et al. 2007 [12]	Brain metastasis in renal cell cancer responding to sunitinib	22	4.5
Fabi et al. 2006 [13]	Regression of multiple brain metastases from breast cancer with capecitabine	15	2.5
Gounant et al. 2007 [14]	Subsequent brain metastasis responses to epidermal growth factor receptor tyrosine kinase inhibitors in a patient with non-small-cell lung cancer	13	2.6
Thibault et al. 2008 [15]	Regression of brain metastases of renal cancer with antiangiogenic therapy	12	3.1
Hodi et al. 2008 [16]	CTLA-4 blockade with ipilimumab induces significant benefit in a female with melanoma metastases to the CNS	11	2.8

## Discussion

This analysis was based on a systematic literature search where we decided to apply a broad definition of brain metastases related publication. We acknowledge that some of the selected case reports might be subject to debate. Moreover, we encourage authors of relevant publications that might have been overlooked to inform us about their article in order to refine future evaluations. Reference [14] represents one of the case reports with a title clearly indicating the type of publication. References [13], [15] provide examples for case reports that were more difficult to identify at first glance. From our point of view, it would be desirable that all case reports could be identified quickly and easily. Journal editors and publishers should take responsibility in providing unambiguous manuscript titles.

In general, the number of publications on brain metastases has increased continuously (Figure 1). The number of case reports has increased as well, while their proportion remained constant. In this analysis, we focused on citation counts. Articles with high numbers of citations are likely those that impressed other scientists and clinicians, and had more influence on clinical practice or future developments in the field than articles with few citations. However, the majority of case reports published in the time period 2006–2010 received limited attention or were not cited at all. Those reporting on two cases received citation counts comparable to those describing only one case. However, the number of articles reporting on two cases was low. The maximum number of citations was 30 as compared to 282 [17] for non-case-reports published during the same time period. All case reports with 10 or more citations were published in the English language and reported on newly introduced anticancer drugs, which commonly are prescribed to treat extracranial metastases, and the responses observed in single patients with brain metastases. Even if none of the drugs has become a standard of care for patients with brain metastases by now, the initial observations made in these case reports might trigger formal prospective studies or sometimes provide clues towards a therapeutic option for heavily pretreated patients when no more standard of care exists. Of course publication bias must also be considered. One cannot exclude that the same drug may have been tried in individual cases in lots of different hospitals, but only the one successful attempt has appeared in the literature. All case reports published in 2009 or 2010 consistently received less than 5 citations. Thus, our results are consistent with the assumption that citation rate is gradually increasing for approximately 2 years after publication. An earlier study evaluated 2-year citation count of case reports published in 1991 and 2001 irrespective of topic [18]. The median citation count was 0 and 1 for case reports published in 1991 and 2001, respectively. Less than 1% received more than 10 citations within 2 years.

As stated previously, we also evaluated average annual citation rate because the exact time course or kinetics of citation is hard to predict and varies with topic and journal [19], [20]. Both accumulation of citations of recently published articles and reduced interest in older articles over time pose challenges if reliable quantitative analysis is attempted. We did not account for date of publication, i.e. whether an article was published earlier or later during a given year. For the purpose of this study, the chosen methods are sufficient. Of course, more detailed and quantitative analyses can be performed with the internet based tools available. It should be noticed that searches in different databases will result in somewhat different citation counts and that the present results (based on Scopus) therefore provide only a snapshot. Self citation is likely to influence the final citation count of sparsely cited articles, whereas its impact on highly cited articles might be less pronounced. It was recently estimated that 6.4% of all citations per article (interquartile range 2.8–11.3, mean 8.4) were self citations [21]. Studies most vulnerable to this effect were those with more authors and small sample size. However, this study did not focus on case reports.

In conclusion, research activity has increased in the time period between 2006 and 2010 and case reports continue to contribute approximately 20–21% of all publications. It cannot be excluded that case reports without citation provide interesting information to some clinicians or researchers, but their ultimate role is difficult to quantify. Apparently, case reports describing unexpected therapeutic success gain more attention, at least in terms of citation, than others.
